# The wear and tear of racism: Self-silencing from the perspective of young Black women

**DOI:** 10.1016/j.ssmqr.2023.100268

**Published:** 2023-04-10

**Authors:** Jewel Scott, Kortney Floyd James, Dara D. Méndez, Ragan Johnson, Esa M. Davis

**Affiliations:** a University of Pittsburgh School of Medicine, Pittsburgh, PA, USA; b University of California, Los Angeles David Geffen School of Medicine, Los Angeles, CA, USA; c University of Pittsburgh School of Public Health, Pittsburgh, PA, USA; d Duke University School of Nursing, Durham, NC, USA

## Abstract

**Context:**

Historically, Black women strategically employed silence to endure enslavement to the U.S., and other forms of racial violence. The current study aimed to understand contemporary perspectives on self-silencing.

**Objective:**

To explore young adult Black women’s experiences of self-silencing and its potential impact on their physical and mental well-being.

**Methods:**

Data are from 16 semi-structured interviews with Black women ages 18 to 39 in southwest Pennsylvania conducted between October 2021 - May 2022. We analyzed the interviews using inductive thematic analysis.

**Results:**

We identified four themes: “Self-silencing is Inherited,” “Silencing Here and Now,” “Wear and Tear,” and “The Flip Side.” The first theme represents the overwhelming consensus that limiting self-expression has a generational component rooted in racism. Most participants identified self-silencing in school and employment settings. Participants described the wear and tear of self-silencing as negatively impacting health behaviors (e.g., diet) and mental health both when deciding whether to self-silence and later ruminations on the decision. “The Flip Side” represents counter perspectives that not self-silencing liberates and improves health.

**Conclusions:**

The findings highlight that many Black women may use or resist self-silencing as a vigilance-based coping strategy to preserve their mental and physical well-being. We present measurement considerations for research on health impacts of racism and other forms of oppression.

## 1. Introduction

“When we speak we are afraid our words will not be heard or welcomed. But when we are silent, we are still afraid. So it is better to speak.” Audre Lorde

Racism is a fundamental cause of health inequities, impacting mental and physical well-being, that currently and historically affect Black people in America since their arrival to the U.S. in 1619 (Bailey et al., 2017). In addition, the intersection of having at least two historically oppressed identities, ‘race’ and gender, exposes Black women to gendered racism (Vance et al., 2022). Among Black women, racism is associated with high blood pressure, depression, obesity, preterm birth, and subclinical cardiovascular disease (Abrams et al., 2019; Himmelstein et al., 2015; Jakubowski et al., 2021; Lee & Hicken, 2016; Mendez et al., 2014; Williams et al., 2019). However, refining the measurement of racism is often identified as a methodological limitation of existing research (Adkins-Jackson et al., 2021; Williams et al., 2019). Conceptualizing racism as “a pervasive system of power,” as the American Public Health Association and others do, suggests a need to capture the effects of racism related to the power differentials that disadvantage people of color who are deemed inferior because of the “social interpretation of skin color ”((APHA), 2021; Jones, 2000). Racism as a pervasive oppressive system of power was and still is maintained by the threat of violence, including lynching and excessive policing, which shapes how Black women respond to racism (Wilkerson, 2020).

To survive racism, Black women used a self-silencing strategy to endure being kidnapped and enslaved to the U.S., and physical, sexual, and emotional violence; and many continue to rely on this strategy to endure contemporary manifestations of racism such as police brutality and job discrimination (Jacobs, 1860; McKay, 2002; Reilly, 2022; White, 1999). Narratives from enslaved women indicate the decisive use of silence in harsh circumstances, such as an elderly enslaved woman being sold at auction despite decades of promises of freedom upon the death of the plantation owner, “Without saying a word, she quietly awaited her fate. At last, a voice said, ‘fifty dollars’ ”, or commands to demonstrate their value, “Show your neck Betsey. There’s a breast for you; good for a round dozen before she’s done childbearing” (White, 1999). The forced physical and sexual labor of enslaved women is also widely documented in historical accounts, with the price of resisting or speaking up, even identifying the father of their biracial child punishable in myriad horrific ways (Jacobs, 1860; McKay, 2002). Other historical and contemporary works document workplace and social environments where Black women “shift” or “submerge” aspects of their identity, a variation of silencing their true selves, to conform to expectations or resist stereotypes of being angry or assertive (Rosette et al., 2018; White, 1999). The burdensome use of silence was recently displayed in the Fall of 2022 as an intoxicated, young White woman hurled racial slurs at a young Black peer who was working as a desk clerk in a university dormitory (Reilly, 2022). While some praised the Black woman’s “restraint and calm”, and many on social media lamented the experience on her behalf, the young Black woman describes the ordinariness of the experience as an “recurring issue” in America (Reilly, 2022). The strategic use of silence, not speaking in moments often of injustice and duress, is a common coping strategy used in the lives of many Black women and has been underexplored in the health science literature.

Self-silencing is defined as the tendency to limit self-expression or speaking, often to avoid conflict, preserve harmony, or maintain relationships and was initially conceptualized as a way to understand the high prevalence of depression among women (Baeza et al., 2022; Jack & Ali, 2010). The limited health sciences research focused on self-silencing identified associations with depression, subclinical cardiovascular disease, mortality, and HIV-risk behaviors (Abrams et al., 2019; Eaker et al., 2007; Hurst & Beesley, 2013; Jakubowski et al., 2021; Stokes & Brody, 2019). Specific to Black women, a recent study found an association of self-silencing with atherosclerotic cardiovascular disease among middle-aged Black women, but not White women (Jakubowski et al., 2021). More research is needed to understand Black American women’s perspectives and use of self -silencing and how it affects their health.

To address racism as a public health crisis, research is needed to refine the conceptualizations and measurement of racism and the myriad ways that racism impacts the health of Black people (Hardeman et al., 2022; Jones, 2000; Mendez et al., 2021). An examination of self-silencing, from the perspective of young Black women, is an opportunity to reflect on the historical use of silence and understand Black women’s contemporary experiences of self-silencing. The current study aimed to explore young adult Black women’s perspectives, beliefs, and experiences of self-silencing in their daily lives and its potential impact on their physical health and mental well-being.

### 1.1. Theory

Dana Crowley Jack’s Silencing-the-self theory originates from qualitative research with women across different cultures and observing the lack of power as a unifying theme (Jack & Ali, 2010). She hypothesized that feelings of powerlessness led to more emotional suppression contributing to women’s greater vulnerability to depression than men. Consistent with the theory, a recent concept analysis identified fear, powerlessness, and self-judgment as antecedents to self-silencing, and provides a case example of self-silencing among Latinas due to cultural expectations of submissiveness (Baeza et al., 2022). However, other than research with immigrant Black women of Caribbean descent living in the U.S. (Ali, 2010), there is a paucity of research that examines the nuanced ways that self-silencing may be relevant to the lived experiences of Black women.

The primary investigation of self-silencing as used by Black women connected the strategic use of silence with many Black women’s endorsement of the Strong Black Woman or Superwoman Schema (Abrams et al., 2019). This construct encompasses the many ways Black women armor themselves to survive the stressors related to being both Black and a woman; an intersection of social identities that situates Black women at the bottom of the social hierarchy (Jordan-Zachery, 2007; Woods-Giscombe, 2010). Abrams et al. (2019) identified that self-silencing partly explains how suppressing emotions and experiences to fulfill the obligation to be strong contributes to depressive symptoms among Black women. Another study with college-aged women identified that self-silencing partly explained the harmful impact of sexism on psychological distress (Hurst & Beesley, 2013). The connection of self-silencing with mood is consistent with self-silencing as a gendered view of depression (Abrams et al., 2019; Hurst & Beesley, 2013; Jack & Ali, 2010). Other research explores self-silencing specific to sexual health and HIV risk behaviors (Stokes & Brody, 2019). Building the literature documenting the physical health consequences of self-silencing requires a deeper understanding of the lived experience of self-silencing among Black women.

## 2. Methods

The research team consisted of five self-identified Black women (three doctoral prepared nurse researchers, one physician researcher, and one social epidemiologist) trained in qualitative methods. Peer debriefing was implemented in the conceptualization of the study, before coding and throughout data analysis to support our reflexive practice and enhance rigor and trustworthiness of study findings (Glesne, 2016; Vaismoradi et al., 2013).

### 2.1. Recruitment

From October 2021 to May 2022, we used community-based recruitment (e.g., support groups for Black mothers) to invite Black women in southwestern Pennsylvania to participate in a research interview. Details provided in advertisements can be a source of sampling bias, inadvertently attracting one group or unintentionally excluding another group (Glesne, 2016). The advertisements for the research study sought volunteers for an interview on the “social experiences and communication practices of young adult Black women”. Paper and electronic advertisements were distributed to employees of a Black-led non-profit organization (home visiting program for new mothers), around the local college campus, emailed to the presidents of Black sororities, and face-to-face in the parking lot of grocery and beauty supply stores. The flyer was also placed on a social media support group for Black mothers in the local area and distributed via a group text app coordinated by students of color (e.g., GroupMe). Inclusion criteria were: 1) self-identify as Black or African American, 2) self-identify as female, 3) 18–39 years of age, 4) have access to internet services to use videoconference technology, and 5) fluent in English. The age range was restricted to include only young Black women to understand contemporary experiences of self-silencing.

### 2.2. Procedure and data collection

The study was approved by the institutional review board and informed consent was elicited before the interview. The principal investigator (PI) conducted all interviews via videoconference technology using a semi-structured interview guide. The interviewer kept her camera on to promote connection and rapport with participants; participants chose to have their camera on or off based on their level of comfort. Participants selected a pseudonym to be used during the interview to protect their confidentiality. The interview guide started with a broad question to elicit participants’ initial thoughts about self-silencing. Next, the study PI read a standardized definition of self-silencing to the participant; the PI probed for any additional thoughts. The remainder of the interview guide funneled down by asking about personal experiences and the experiences’ impact on participants’ physical and mental health. The following questions were covered in the interview: 1) Are young adult Black women aware of “self-silencing” and what are their thoughts about self-silencing as a concept?; 2) How does “self-silencing” relate to their life?; and 3) What are young adult Black women’s thoughts on how self-silencing affects their physical and mental health?

On average, interviews lasted 44 min. After the interview, respondents completed a demographic questionnaire and received a $35 visa card for study participation. Interviews were recorded and professionally transcribed verbatim and the PI reviewed transcripts for accuracy.

### 2.3. Data analysis

Thematic analysis using inductive coding was appropriate for the study’s aim to extract commonalities that are then grouped into themes(Clarke, Braun, & Hayfield, 2012; Vaismoradi et al., 2013). Data analysis began after ten interviews were completed; interviews continued in parallel with analysis. Data were analyzed using Dedoose^©^ Version 9.0.46 (SocioCultural Research Consultants, 2021). After no new codes/themes were identified, several more interviews were conducted to confirm saturation, resulting in a total of 16 complete interviews. Because the eligibility criteria yielded a sample with similar characteristics, saturation was achieved quickly. Data saturation is often reached with 9–17 interviews when the study has a relatively homogeneous study population (Hennink & Kaiser, 2022).

Three investigators familiarized themselves with the data by reading and rereading transcripts multiple times and independently labeled pertinent features of several transcripts to generate initial codes (Vaismoradi et al., 2013). We shared our independent analysis, identifying areas of agreement and discussing codes that were different. Through discussion, rereading the data, and cycles of comparison, we came to agreement on the coding. However, additional codes were added as needed, and data was coded and re-coded as the analysis process unfolded (Clarke et al., 2012; Vaismoradi et al., 2013). We continued through the recursive process of phases three through five of thematic analysis (i.e., search for themes, review possible themes, and define and name themes) with the entire research team (Clarke et al., 2012). Sub-themes were grouped into themes of self-silencing, and the report was generated in parallel with the evolving naming of themes (Clarke et al., 2012; Vaismoradi et al., 2013). Lastly, we sought feedback from the participants to affirm the themes and quotes accurately reflected their experiences by emailing a summary of the themes and exemplar quotes to each participant. Two participants responded and expressed their agreement with the themes.

## 3. Results

Characteristics of the fifteen participants that completed the demographic questionnaire are provided in Table 1. One participant did not complete the questionnaire. Seven (46.7%) of the participants were college educated, six (40%) were full-time students, and three (20%) were married. The following medical conditions were self-reported: depression (N = 5, 33.3%), anxiety (N = 4, 26.7%) and obesity (N = 4, 26.7%); five women (33.3%) reported no past medical history. Table 2 provides representative quotes for the four identified themes: Self-silencing is inherited, Silencing here and now, Wear and tear, and The flip side.

### 3.1. Self-silencing is inherited

*Self-silencing is inherited* represents the perspective of many of the participants that withholding opinions and feelings is a trait that Black women “inherited” from past generations to survive. “*We weren’t expected to have a voice*”, exclaimed one participant. Taylor shared that “*Black women in our history were arrested for speaking up. Their children were taken away*.” as an example of how racism has made some Black women “*reluctant to speak*”.

Most participants described how they have suppressed their voice often and throughout their life. There was a common understanding among participants that all women, regardless of race, may self-silence, but that its commonness is far greater for Black women living in America. For example, Peanut noted “*the severity of what could happen is different, like we [Black women] have more at stake*”. When recounting the origin of their self-silencing behavior, some participants attributed it to being passed down from their enslaved ancestors who used self-silencing to survive, others could not pinpoint how they acquired the ability to self-silence, and others described parents and elders as role models who demonstrated or enforced self-silencing. For example, a participant commented that “*it was definitely taught to me*” and another, Peanut, described having conversations with her school-aged daughter because the daughter says “*whatever comes to her mind*” and she worries about the consequences of her daughter being “*expressive*”. This theme highlights the historical and transgenerational contributors to why some Black women not only suppress their voice, but also have learned when to withhold their perspective. The participants in this study understood the generational influence of self-silencing started in early childhood and has ramifications for their daily life.

### 3.2. Silencing here and now

*Silencing Here and Now* reflects participants’ contemporary experiences with self-silencing and their momentary awareness of silencing. Often participants recounted experiences in their professional lives, whether place of employment or classroom setting. For some participants, self-silencing at work was tied to their obligations to “*maintain my job, because I have this kid*”, describing a need to provide for their family. They also lamented their decision and wondered “*do I continue to go home every day thinking like, oh man I wish I said something”*.

The participants’ also shared that their marginalized identities (i.e., being Black and a woman) was often at the core of their decision to silence, specifically using silence out of fear that their behavior would conform to a stereotype (i.e., stereotype threat). Participants described living with a persistent awareness of the judgments, labels, and stereotypes imposed upon Black women by the dominant society. They anticipated racial discrimination, such as being labeled “*angry*”, “*loud*”, or “*ghetto*”, and preemptively adjusted their behavior. One participant described her tendency “*to make myself a lot quieter than I actually am*” because she does not want to be “*the loudest one*”. Others observed that they silence their response when they have experienced racism. Gail described it feeling “*like a slow-mo, but [also] like a quick 5 s”* while she is processing how to respond. Participants described considering “*is it worth the energy*”, “*do I risk losing my job?*”, “*Is this really happening right now? What’s my next step?”*. Many participants reflected on the mental calculations Black women make in the moment when deciding whether to self-silence, sometimes noting the toll these internal second-guessing exercises take on their “*mental and spiritual energy*”. The participants described self-silencing as an active decision made at the moment, but the mental anguish of not speaking extended beyond the immediate moment.

### 3.3. Wear and tear

The Wear and Tear theme is drawn from The Weathering Hypothesis which posits that Black women experience premature declines in health related to chronic racial stress (Geronimus, 1992). The Black women in this study described the health consequences of regularly self-silencing since a young age using phrases such as “*over time*”, “*eventually … it weighs on your physical health*”, and “*I essentially would become like a ticking time bomb*” to explain their perceptions of the cumulative effects self-silencing has on their health. The wear and tear on mental health was sometimes attributed to the need to “*stay on your feet*,” to decide whether to self-silence. Many participants described later revisiting their decision to not speak, described feeling stressed or anxious about the decision, and coped with this complex emotional response through food and other behaviors. Tiffany connected changes in their mood due to self-silencing with disruptions in their diet or physical activity, noting “*You’re depressed. You’re going to just overeat or under-eat. But that comes from holding stuff in or not expressing yourself*.” Other participants described eating as a source of comfort for the emotional pain related to self-silencing with statements such as “*eating gives me peace. It makes me feel good. It’s a temporary relief, but it feels good*.”

The wear and tear on their health also referred to how Black women engage when in healthcare settings. Most often, participants described self-silencing in anticipation of a discriminatory experience with the healthcare provider, specifically when being ignored or actively silenced by their provider. Some participants chose not to speak with healthcare providers about their symptoms or experiences because they did not feel they would be believed. Other participants described being directly silenced by healthcare providers “talking over” them or not providing information in a way that is easy to understand.

### 3.4. The flip side

The Flip Side illustrates alternative perspectives of self-silencing. While earlier themes described the oppressive or mentally taxing aspects of silencing, other participants decided “*I don’t silence myself anymore … I got tired*” describing their movement from self-silencing to not silencing. Seeing an older Black woman speak up was empowering for another participant and she remembers thinking, “*Wait a minute. … they [employers] have to respect you?*” For her, the modeling of an alternative to self-silencing was a pivotal moment. For others, they moved to the flip side because “*[in] therapy I get to talk about all the stuff that I silence myself about*”. Sometimes, the participant described making decisions based on the person’s openness to having dialogue, because “… *it’s about preserving myself*”. Similarly, another participant described using an “*active silence*” to listen but distinguished that from the “*avoiding silence*” that drained her health; “*Now, I’m not numb. I feel things.. I react to them … I move with intention.*” Participants described purposeful changes in their self-silencing behavior that felt beneficial to their health and wellbeing.

## 4. Discussion

In this qualitative study, we explored how young Black women applied the concept of self-silencing to their lives. Four themes emerged from our study that highlights the temporal development of self-silencing in the lives of young adult Black women, the context when silencing regularly occurs, and the perceived toll of self-silencing on their physical and mental health. Our results indicate that self-silencing is a complex experience for Black women and may develop in response to racism and/or in anticipation of a racist encounter. Findings from the current study contributes to the growing literature on the impact of structural and interpersonal racism on health and can inform future conceptualizations and measurements of racism in research.

There was an overwhelming consensus among the Black women from our study that self-silencing was generational. Many women shared that they either witnessed their mothers self-silence or were taught by other family members to self-silence at an early age. Further, in the U.S. self-silencing was modeled or actively taught by Black mothers to their daughters as a means of safety and survival when living in a race-conscious society. Previous research highlights the parenting practices of Black families (e.g., instructions regarding how to interact with police, instilling Black pride, assimilation) that protect and/or prepare their children for interpersonal and structural racism (Jackson et al., 2017; Lewis, 2019; Sewell et al., 2016). This study expounds on previous findings by focusing on the cultural norm of self-silencing in Black women as a cross-generational strategy to avoid and/or respond to experiences of racism.

Racism as a chronic stressor in the lives of Black Americans is often conceptualized as a response to interpersonal mediated racism. We observed that self-silencing to avoid identification with a racist stereotype (i.e., stereotype threat) is also common among Black women even if they have no direct experience with the stereotype, for example, they have not personally been called ‘angry’ or ‘loud’. The current study extends the existing research by identifying that many Black women silence themselves as a behavioral response to stereotype threat, cultural and structural racism (Hicken et al., 2019).

Their later rumination about a racist event suggests an active suppression of emotion in the moment, which creates inner turmoil to be reflected on at a later time. This finding may relate to the body of research on emotional suppression and its effect on the stress-responsive hypothalamic-pituitary-adrenal (HPA) axis. Research on emotional suppression suggests that negative emotions, such as those generated when self-expression is restricted, may contribute to heightened inflammation, but to our knowledge this has not been tested among Black women (See Renna 2021 for a review of negative emotions and inflammation) (Renna, 2021; Sawyer et al., 2012). However, Jakubowski and colleagues examined the association of atherosclerosis, a disease of inflammation, with self-silencing (Jakubowski et al., 2021). Findings of greater carotid atherosclerosis among middle-aged Black women who endorse self-silencing, but not among White women, provides preliminary support for this hypothesis. Further research is needed to explore emotional regulation as a possible pathway that explains how self-silencing due to racism harms the health of Black women.

The women in this study identified decreased engagement in health behaviors as an example of how self-silencing affects their physical health. The behaviors that women associated with self-silencing including increased consumption of comfort foods (e.g., binge eating) and decreased physical activity are prime health behaviors for maintaining health and for the prevention of cardiovascular disease and other chronic diseases (Goode et al., 2020; Kang et al., 2014; Virani et al., 2020). Their examples resonate with the literature on food-based coping and health outcomes among Black women (Diggins et al., 2015; Harris et al., 2021; Reid et al., 2016; Woods-Giscombe et al., 2021). Food-based coping is common among Black women, with 93% of the 189 Black women in a recent study endorsing using food to cope with stress, and food-based coping was associated with higher BMI (Woods-Giscombe et al., 2021). Another study of 99 Black college women also found associations between higher BMI, race-related stress, and emotional eating (Diggins et al., 2015). The relationship between food-based coping and self-silencing has not been evaluated, but one study explored the impact of self-silencing on physical activity. No relationship was identified between self-silencing and physical activity in the sample of 28 Black women with a mean age of 47 (Banks & Xu, 2013). However, self-silencing may decrease as Black women mature and become more comfortable with using their voices to address racism, suggesting a need for examining self-silencing in adolescence and young adulthood.

Participants also described the wear and tear of self-silencing on their mental health, specifically noting symptoms of depression and/or anxiety. This observation aligns with the origins of self-silencing theory as a way to understand the higher prevalence of depression among women (Jack & Ali, 2010). Our research adds an intersectional perspective by centering on Black women’s experiences of gendered racism (Lewis et al., 2013; Vance et al., 2022), revealing a use of self-silencing not well described in the health science literature. Some participants described self-silencing in anticipation of a racist encounter, which corresponds with public health research exploring vigilance as a response to racism (Hicken et al., 2019; Lee & Hicken, 2016). Vigilance, in the form of self-silencing, was often part of their process in deciding how to ‘show up’ in classroom and employment spaces where their lack of power was shaped by their race, gender, and other intersectional identities. Among 3000 adults in Chicago, Black adults reported worse sleep quality than White adults, and this was mostly mediated by racism-related vigilance (Hicken et al., 2013). An analysis of the data from the 1200 Black adults in the same study identified anticipatory vigilance, including restricting speech, was associated with more depressive symptoms (Lee & Hicken, 2016). Specific to young Black women, analysis of data from 69 Black college women reported similar findings with a greater tendency to ruminate about stressful events, mediating the relationship between race-related vigilance and more depressive symptoms (Hill & Hoggard, 2018). Findings from our study highlight that young Black women may use or intentionally not use self-silencing as a vigilance-based coping strategy to preserve their mental and physical well-being.

### 4.1. Strengths and limitations

The qualitative nature of this study was for in-depth exploration of perceptions and thoughts, views of a purposive sample of young Black women in a northeastern city. Although this study is limited to a specific group of Black women, this study adds to our understanding of an important phenomena, self-silencing, in response to racism and sexism. Based on the shared history and the prolific violence of racism across the U.S., and especially in the southeast, we hypothesize that self-silencing may be a part of the daily realities of many Black women across the country. Also, a third of participants self-reported mental health diagnoses. We are unable to identify an aspect of recruitment that would have attracted more women with a mental health diagnosis. However, we suspect that given the prevalence of mental health diagnoses in women, this may reflect the mental health crisis in Black America that is partly due to coping with racism (Bor et al., 2018; Jackson et al., 2017).

Our findings highlight a need for research to examine self-silencing as a vigilance-based coping strategy, and its potential negative associations with diet, physical activity, and sleep behaviors among young Black women (Table 2, column 3). Ruminating and “cognitive imagery” of a negative experience, such as racism, can prolong the negative health consequences and also triggers more vigilance (Lewis et al., 2015). A focus on young adult women, and potentially adolescents, is essential because the foundations of health and disease are established years before disease onset. Also, research exploring the transgenerational consequences of self-silencing, including biological processes related to the embedding of stress, is needed. Our findings indicate that self-silencing may be a variation of racism-related vigilance, an area that is underexplored in the published literature on racism and health, and in need of measurement development. Additional key areas for further study include research to examine the biological pathways connecting self-silencing, physical and mental health and determining the short- and longer-term health effects of disrupting the transgenerational transmission of self-silencing behavior (Table two, column three).

Equally important, is research to determine whether the mental and physical impacts of self-silencing are reversed for Black women who do not self-silence or who have a strong racial identity. An important step would be applying a community-engaged approach to support Black women in using their voices as power (e.g., resistance-based coping) (Lewis et al., 2013) and as a way to protect their health and wellbeing. Additionally, this work would need to be combined with a multilevel approach to change the institutions and systems responsible so that the adaptive behavior of self-silencing is no longer needed. The removal of the vigilant behavior of Black women in a society where anti-Black racism continues leaves them vulnerable to the violence of racism (Jackson et al., 2017; Wilkerson, 2020); however, continuing the vigilant behavior will be detrimental to both their dignity and humanity.

## 4.2. Conclusion

The participants in the current study indicate that Black women decide to self-silence in the moment to prevent physical, mental, or emotional harm. Our findings highlight the chronicity of self-silencing among Black women and the momentary awareness of the mental stress and energy required to frequently silence to meet the need for psychological safety. However, deciding to freely express one’s voice can feel liberating and powerful for these participants. The rich descriptions of silencing in anticipation of racism, such as stereotype threat, support existing literature on vigilance-based coping. We identified opportunities for refining the conceptualization or racism and its impact on the health of young Black women.

## Figures and Tables

**Table 1:** Sample characteristics (N = 15).

Age	*f* (%) or M ±SD
	26.1 ± 7.4 (18–37)

Race (Black or African American)	15 (100)
U.S. Citizen	
Yes	14 (93.3)
No response	1 (6.7)
Marital status	
Single	12 (80.0)
Married or partnered	3 (20.0)
Income	
< 20,000	4 (20.0)
20,000–49,999	5 (33.3)
≥50,000	4 (20.0)
Employment	
Full-time student	6 (40.0)
Full-time employment	5 (33.3)
Part-time employment or not employed	3 (20.0)
Self-reported history of diagnosed medical condition	
Depression	5 (33.3)
Anxiety	4 (26.7)
Obesity	4 (26.7)
Post-traumatic stress disorder (PTSD)	2 (13.3)
Autoimmune diseases	2 (13.3)
Hypertension	1 (6.7)
Endometriosis	1 (6.7)
Other	1 (6.7)
None	5 (33.3)

**Table 2:** The four themes and exemplar quotes identified in the thematic analysis^a^.

Themes	Exemplar Quotes	Research Implications
**Self-silencing is inherited**	“Self-silencing is a very normal part of Black women’s lives, and you don’t realize it until you talk to other people, of other races a lot of times. So, I think self- silencing can be universal in some sense. I don’t know if anybody else does it to the degree that Black women do, at least in America.” [Jan, age 20] “We weren’t expected to have a voice. So sometimes communication and speaking up was just amongst ourselves, nothing publicly. Because being outspoken was looked at as being aggressive. It was looked at as being a troublemaker. Black women in our history were arrested for speaking up, jailed. Their children were taken away. Black women were taken away from their children. So many things happened. I think Black women were forced to be reluctant to speak up.” [Kim, age 36] “The whole, “I’m going to make you scared of me if you speak up” type of thing, the repercussions and punishment for saying things … that did a number. That definitely impacted my ability to competently speak on things without a fear of repercussion.” [Amy, age 18]	How to disrupt the transgenerational transmission of vigilance; Does disrupting the transgenerational transmission of vigilance create greater harms for the next generation?
**Silencing in the Here and Now**	“. if you are used to not being heard or used to it not making a difference when you do speak up, then you begin to just self-silence yourself. And I feel like, for white women, they’re more likely to be taken seriously, they’re more likely for something to be done if and when they do speak up. So, they’re more likely to not silence what they feel. And then also dealing with, “Will I be portrayed as a loud black woman or a black woman with an attitude?” Not wanting to be portrayed negatively when I think a white woman would just be portrayed as assertive, we might be portrayed as loud and ghetto or difficult.” [Natra, age 37] I’ts a tough line because I understand it [self-silencing] is not good and is probably not healthy, but at the same time, I think unemployment is also not healthy … Sometimes i’ts a safety thing you know, so do I risk losing my job for speaking back … or do I continue to go home every day thinking like, oh man I wish I said something I think i’ts a struggle for a lot of people and, and if you’re not in a position where you’re in power most Black women we are not in those positions. So, sometimes, we do have to take the disrespect to take these prejudices and biases in order just to get the job done.” [Jan, age 20] “Well, am I overreacting? Am I misconstruing what was being said? Or maybe I’m the onejustblowing it out of proportion. I need tojust let it go, depending on what the situation is … Maybe I should make myself even smaller.” [Keshia, age 32]	How does the body respond to racism induced anticipatory stress such as vigilance and negative emotionality (e.g., anger, fear)
**Wear and Tear**	“You’re depressed. You’re going tojust overeat or under-eat. But that comes from holding stuff in or not expressing yourself or feeling like you’re not good enough or you’re not understood. So, it can definitely tap into your physical health, not wanting to be active.” [Tiffany, age 28] “I guess i’ts like stress. I feel like stress and kind of just being depressed or being anxious is bad for your physical health, like blood pressure, heart rate. I know my heart rate is usually pretty high just because I’m stressed out for no reason half the time … a domino effect, I guess. ” [Brooklyn, age 21] “People are speaking over us and to us about what they’re going to do. The choices are taken away and we don’t even understand. Half the time i’ts not broken down to us into language that we understand. And so, I feel like I’ve never seen self-silencing so largely than in a birthing room …” [Natra, age 37]	Adaptive coping in response to stress Impact of self-silencing on the hypothalamic-pituitary-adrenal (HPA) axis Depression as a risk factor for declines in physical health
**The Flip Side**	“The older lady at my job … and my boss. They got into it in front of all of us. And [the older lady] was like, “No, you’re going to respect me. You’re going to address me by my name. I’m not a ‘her’. I have a name.” … he [our boss] apologized to her, and he called her by her name. And I was like, “Wait a minute. You can say that to your boss? And they have to respect you?” I was like, “Oh, man. What have I been doing wrong?” [Roxy, age 31] “Based on the conversation on hand, I can understand where you are … in hearing what I’m saying. So I know how much energy I need to put into it. … I’ts about preserving myself” [Mia, age 32] I don’t know if there’s a difference between silences … the active silence, I’m not just not speaking, I’m not speaking to listen. So, the times where I’m listening is a good silence. If I’m just like, being quiet for the sake of not having drama, then i’ts kind of not really helpful.” [Peanut, age 34]	Differential impact of self-silencing on health Reversal of health impacts of racism and self-silencing Strengths-based approaches to health

a All names are pseudonyms.
